# Dietary butyrate alleviates high-fat diet-induced colonic mucosal impairment accompanied by increased gut-derived indole-3-acetic acid and enhanced PPARγ signaling

**DOI:** 10.3389/fendo.2026.1971187

**Published:** 2026-10-21

**Authors:** Guoli Li, Sijing Dong, Chunhao Liu, Huan Yan, Jing Yang, Patrick C.N. Rensen, Bingyin Shi, Yanan Wang

**Affiliations:** 1 Department of Endocrinology, First Affiliated Hospital of Xi’an Jiaotong University, Xi’an, China; 2 MED-X institute, Center for Immunological and Metabolic Diseases, the First Affiliated Hospital of Xi’an Jiaotong University, Xi’an, China; 3 Department of Medicine, Division of Endocrinology, and Einthoven Laboratory for Experimental Vascular Medicine, Leiden University Medical Center, Leiden, Netherlands

**Keywords:** butyrate, gut barrier, gut microbiota, indole-3-acetic acid, obesity

## Abstract

**Introduction:**

High-fat diet (HFD) induces intestinal barrier impairment, associated with gut microbiota dysbiosis, endotoxin translocation and metabolic dysfunction. Dietary butyrate has been reported to improve intestinal homeostasis, but the underlying mechanism remains incompletely understood. This study aimed to investigate the effects of dietary butyrate on HFD-induced colonic mucosal dysfunction and to explore underlying mechanisms with respect to the role of gut microbiota and its metabolites.

**Methods:**

Male APOE*3-Leiden.CETP mice, a well-established translational model for diet-induced metabolic dysfunction, were fed an HFD with or without 5% sodium butyrate. Metabolic phenotypes, plasma lipopolysaccharide (LPS), colonic histology, inflammatory responses, and tight junction-associated changes were assessed. Fecal microbiota transplantation (FMT) was performed in antibiotic-treated recipient mice to assess the contribution and transferability of butyrate-shaped gut microbiota. Cecal metagenomic sequencing and LC/MS-based colonic indole-3-acetic acid (IAA) measurement were performed. Caco-2 cells were used to assess the direct effects of sodium butyrate and IAA on enterocytes and to determine, through pioglitazone treatment and PPARγ knockdown, whether PPARγ mediates IAA-induced enterocyte responses.

**Results:**

Dietary butyrate induced favorable changes in multiple colonic barrier-associated parameters, including improved colonic architecture, lower circulating LPS levels, decreased *Tnf-a* and *Il-1b* expression, and increased CLDN2, Reg-family, and PPARγ expression in the colon. In addition, butyrate reduced body weight gain, fat mass accumulation and improved glucose metabolism. FMT from butyrate-treated donors partially reproduced these beneficial effects in HFD-fed recipients. Metagenomic analysis showed enrichment of *Bacteroidota* and *Bacteroides*, particularly *B. uniformis* and *B. thetaiotaomicron*, accompanied by increased colonic IAA levels. In Caco-2 cells, IAA increased the expression of PPARγ, *Reg4*, and CLDN2. While PPARγ agonist pioglitazone mimicked the upregulation of REG4 and CLDN2 expression by IAA, siRNA-induced PPARγ knockdown attenuated IAA-induced REG4 and CLDN2 upregulation.

**Conclusion:**

Dietary butyrate improves colonic mucosal homeostasis under HFD feeding, accompanied by gut microbial remodeling and increased colonic IAA levels. Together with the PPARγ-dependent epithelial response to IAA observed in Caco-2 cells, these findings suggest a potential mechanistic framework linking butyrate-associated microbial changes, IAA metabolism, and epithelial PPARγ signaling.

## 1 Introduction

The intestine is not only responsible for nutrient digestion and absorption but also serves as a critical interface linking the gut microbiota, mucosal immunity, and host metabolic homeostasis (1, 2). This function depends on an integrated intestinal barrier composed of commensal microbes, the mucus layer, epithelial cells connected by tight junctions, antimicrobial peptides, and mucosal immune components (2, 3). Under physiological conditions, this barrier permits nutrient uptake while restricting the translocation of luminal bacteria, antigens, and endotoxins. However, high-fat diet (HFD) disrupts intestinal barrier integrity by altering gut microbial ecology, impairing tight junction organization, increasing epithelial stress and inflammatory signaling, and facilitating lipopolysaccharide (LPS) leakage into the circulation (4, 5). Thus, HFD-induced gut barrier dysfunction may represent an important contributor to metabolic inflammation and metabolic impairment.

In contrast to HFD, a diet rich in dietary fiber provides polysaccharides for gut microbiota fermentation, producing short-chain fatty acids (SCFAs) (6). Among SCFAs, butyrate has been extensively reported to attenuate HFD-induced obesity and improve glucose metabolism in rodents (7). Additionally, butyrate modulates the intestinal immune microenvironment and reinforces epithelial barrier integrity (8). In experimental colitis models without HFD feeding, butyrate has been shown to suppress intestinal inflammation by promoting macrophage polarization toward an anti-inflammatory M2 phenotype and regulating the function of regulatory T cells (9, 10). *In vitro* studies further indicate that butyrate facilitates tight junction assembly through activation of AMP-activated protein kinase (AMPK) and Akt signaling pathways, thereby enhancing epithelial barrier function (11). However, whether dietary butyrate can ameliorate HFD-induced intestinal barrier impairment, and through which underlying mechanism, remains incompletely understood. Beyond its direct effects on intestinal epithelial and immune cells, butyrate may also exert barrier-protective effects by reshaping the gut microbiota and its metabolites (12, 13).

In fact, gut microbiota has been described to convert dietary tryptophan into multiple indole derivatives, which have been increasingly recognized as important microbial metabolites involved in mucosal homeostasis (14). Given that butyrate can alter gut microbial composition and metabolic activity, it may also influence microbiota-dependent indole metabolism. These gut-derived indole metabolites have been implicated in reinforcing epithelial barrier integrity, modulating inflammatory responses, and maintaining intestinal immune balance (15–17). Therefore, microbial indole metabolites may represent a potential mechanistic link between dietary butyrate supplementation and improved intestinal barrier function. However, whether dietary butyrate enhances the production of gut-derived indole metabolites under HFD-induced obesity, and whether these metabolites contribute to butyrate-mediated gut barrier protection, are rather obscure.

To address these gaps, the present study aimed to investigate whether dietary butyrate supplementation could alleviate intestinal barrier impairment in HFD-induced obese mice. To this end, we evaluated the effects of butyrate on gut barrier integrity, metabolic disturbance, intestinal inflammation, and gut microbial composition. Furthermore, we explored whether butyrate-induced changes in the gut microbial ecosystem were accompanied by alterations in microbiota-derived indole metabolism, thereby identifying potential microbial metabolites associated with butyrate-mediated barrier protection.

## 2 Materials and methods

### 2.1 Animal experiments

Heterozygous APOE*3-Leiden.CETP mice were generated by crossing hemizygous APOE*3-Leiden mice with homozygous human cholesteryl ester transfer protein (CETP) transgenic mice, as previously described (18). Male APOE*3-Leiden.CETP mice were maintained in individually ventilated cages under controlled environmental conditions, with a temperature of 22 °C, relative humidity of approximately 40%, and a 12-h light/12-h dark cycle. At the end of the experiments, mice were weighed and deeply anesthetized by intraperitoneal injection of sodium pentobarbital (100 mg/kg). After adequate anesthesia was achieved, blood samples were collected from the portal vein and heart for plasma preparation. The mice then underwent cardiac perfusion with ice-cold PBS for 5 min, after which the required organs and tissues were collected for further analysis. All animal procedures were performed in accordance with the National Institutes of Health guidelines and were approved by the Animal Care and Use Committee of Xi’an Jiaotong University.

This study included three independent animal experiments.

In the first experiment, 10-14-week-old male APOE*3-Leiden.CETP mice were fed a high-fat diet (60% kcal derived from lard fat and 0.25% cholesterol (w/w), Ready Diets, Shenzhen, China) with or without 5% (w/w) sodium butyrate (Beyotime, #S1539) supplementation for 9 weeks. This cohort was used to evaluate the direct effects of dietary sodium butyrate on HFD-induced metabolic disturbance, colonic barrier-related changes, inflammatory responses, and transcriptomic alterations.

In the second experiment, the contribution and transferability of gut microbiota shaped by dietary sodium butyrate were assessed using fecal microbiota transplantation (FMT). Briefly, recipient mice received 200 μL of an antibiotic cocktail containing ampicillin (0.5 mg/mL), neomycin (0.5 mg/mL), metronidazole (0.5 mg/mL) (all from MedChemExpress, China), and vancomycin (0.25 mg/mL; Macklin, China) by oral gavage once daily for 1 week, followed by antibiotic administration in drinking water for 1 week at half concentrations. Microbiota depletion was verified by fecal DNA quantification. Donor mice were fed an HFD with or without 5% (w/w) sodium butyrate for 3 weeks, and freshly collected fecal pellets were suspended in sterile Ringer’s solution containing 0.05% L-cysteine hydrochloride (Solarbio, China), homogenized, centrifuged, and filtered through a 100 μm cell strainer (Corning, USA). After microbiota depletion, recipient mice were orally gavaged with 200 μL freshly prepared fecal suspension from control or butyrate-treated donors three times per week for 15 weeks and maintained on HFD throughout the experiment. Fecal samples were collected 1 week after FMT to measure DNA concentration and assess recolonization efficiency.

In the third experiment, HFD-fed mice were treated with vehicle or sodium butyrate (4040 mg/kg) by oral gavage daily for 4 weeks to obtain sufficient colonic samples for LC/MS-based measurement of indole-3-acetic acid. The gavage dose was equivalent to that used in the dietary sodium butyrate intervention.

Energy metabolism was evaluated by indirect calorimetry using a light-, temperature-, and humidity-controlled metabolic monitoring system (TSE Systems GmbH, Germany). After 1 week of intervention, mice were individually placed in calorimetric chambers and allowed to acclimate for 1 day before data collection. Oxygen consumption (VO_2_, mL/h) and carbon dioxide production (VCO_2_, mL/h) were continuously monitored, and respiratory exchange ratio (RER) and energy expenditure were calculated accordingly. Locomotor activity was recorded simultaneously. Chamber temperature was maintained at 22 °C for 48 h under a 12-h light/12-h dark cycle.

Body weight was recorded once per week throughout the experimental period. Body composition was assessed before HFD feeding and after every 2 weeks of dietary intervention using a nuclear magnetic resonance-based body composition analyzer (EchoMRI-100; EchoMedical Systems, USA). All measurements were performed at 9:30 AM.

### 2.2 Oral glucose tolerance test and plasma biochemical measurements

Oral glucose tolerance test (OGTT) was performed after 16 h of overnight fasting with free access to water. At 9:00 AM, mice were weighed and orally gavaged with 20% D-glucose at 2 g/kg body weight. Blood glucose was measured from tail-vein blood at 0, 15, 30, 60, 90, and 120 min using a glucometer (Yuwell, China). Glucose tolerance was evaluated from glucose excursion curves using GraphPad Prism v10.

For biochemical measurements in plasma, mice were fasted for 5 h beginning at 8:30 AM. Blood was collected from the tail vein into EDTA-coated tubes and centrifuged at 5,000 rpm for 5 min at 4°C to obtain plasma. Plasma triglycerides, total cholesterol, and glucose levels were measured using enzymatic assay kits Triglycerides (#20767107322), Cholesterol Gen.2 (#03039773190), and Glucose HK Gen.3 (#04404483190) from Roche Diagnostics (Mannheim, Germany) according to the manufacturer’s protocols.

### 2.3 Histology and immunohistochemistry

Colon tissues were fixed in 4% paraformaldehyde for 48 h, dehydrated through a graded ethanol series, and embedded in paraffin. For hematoxylin and eosin (H&E) staining, 5-μm paraffin sections were stained with hematoxylin for 5 min, rinsed with water, counterstained with eosin for 15 s, washed again, mounted with neutral resin, and dried at room temperature. The crypt depth was regarded as the invagination between two villi. Muscular layer width was defined as the thickness of the muscularis externa. For both parameters, three randomly selected fields of identical size were measured for each included sample.

For immunohistochemistry, colonic sections were incubated overnight at 4 °C with the following primary antibodies: rabbit anti-mouse CLDN2 polyclonal antibody (1:100, Proteintech, 26912-1-AP, China), REG3G polyclonal antibody (1:100, ABclonal, A2146, China), REG4 polyclonal antibody (1:200, Proteintech, 12268-1-AP, China), and PPARG monoclonal antibody (1:2000, Proteintech, 81490-5-AP, China). Sections were then incubated with HRP-conjugated secondary antibody (1:2000, Abcam, ab205718, USA), followed by visualization using DAB staining. Slides were scanned with an automated slide scanner (GSscan series, G Cell Technology, China). Integrated optical density (IOD) was quantified using ImageJ software in five randomly selected 100 μm microscopic fields per sample.

### 2.4 Lipopolysaccharide measurement

Plasma LPS concentrations were quantified using a commercial enzyme-linked immunosorbent assay kit (Cusabio, China) in accordance with the manufacturer’s instructions. Briefly, standards and plasma samples were added to the assay plate, and the reaction was developed according to the supplied protocol. Absorbance was measured at 450 nm using a multilabel plate reader (BioTek Synergy Neo2, USA), with wavelength correction at 540 or 570 nm. LPS concentrations were calculated based on the corresponding standard curve.

### 2.5 Colonic transcriptomic analysis

Total RNA was extracted from mouse colon tissues using the TRIzol-based method described above. For library construction, mRNA was enriched with magnetic beads and subsequently fragmented into approximately 200-bp fragments using fragmentation buffer. The fragmented mRNA was reverse-transcribed into cDNA with random primers and reverse transcriptase. After end repair, A-tailing was performed at the 3′ ends of the cDNA fragments, followed by ligation with Y-shaped sequencing adapters. The adapter-ligated fragments were then amplified by PCR and purified to generate the final sequencing libraries. Library sequencing was performed on an Illumina platform using HiSeq X Reagent Kits (Illumina, San Diego, USA). Raw sequencing reads were processed by removing adapter sequences and low-quality reads. The resulting clean reads were aligned to the mouse reference genome GRCm38 using HISAT2. Mapped reads from each sample were assembled with StringTie, and gene expression abundance was quantified as fragments per kilobase of transcript per million mapped reads (FPKM). Differential gene expression analysis was conducted using the limma (v3.60.2) package in R. Gene set variation analysis (GSVA) was performed using the normalized transcriptomic expression matrix and a predefined gene-set file used in the analysis. Enrichment scores for each gene set in each sample were calculated using the GSVA R package (version 1.52.3) with default parameters, and pathways with P < 0.05 were considered significantly altered.

### 2.6 Fecal DNA quantification

Fecal DNA was extracted using the QIAamp Fast DNA Stool Mini Kit (Qiagen, cat. no. 51604) according to the manufacturer’s protocol. Briefly, 180–220 mg fecal samples were homogenized in 1 mL InhibitEX Buffer and centrifuged to remove stool particles. The supernatant was incubated with Proteinase K and Buffer AL at 70 °C for 10 min, followed by the addition of ethanol. The lysate was loaded onto QIAamp spin columns, washed sequentially with Buffer AW1 and Buffer AW2, and DNA was eluted with 200 μL Buffer ATE. Fecal DNA concentration was then quantified using Nanodrop.

### 2.7 Metagenomic profiling of cecal microbiota

Bacterial DNA extraction and metagenomic sequencing were performed with standard procedures as previously described. Briefly, the Magbeads Fast DNA^®^ kit (MP Biomedicals, Shanghai, China) was used following the manufacturer’s protocol to extract bacterial DNA from the stool samples. NanoDrop 2000 was used to measure the concentrations of the extracted DNA, followed by 1% agarose gel electrophoresis for integrity detection using KAPA HyperPlus PCR-free reagent (Illumina, CA, US). DNA was trimmed to an average of approximately 400 bp to construct a paired-end library. Qubit 2.0 (Thermo Fisher, Massachusetts, US) was used to measure DNA quantity, and the reads quality control procedure was conducted using Agilent 5400 (Agilent, Palo Alto, US). Then, pooling was performed on all sample libraries, and the total amount of DNA after pooling was required to be 300 ng, with a volume of 48 µL. After heat denaturation, single-strand circularization, enzyme digestion, and purification, shotgun metagenomic sequencing was performed by Shaan Probiomicros Co.,Ltd. (Xi’ an, China) on the BGI platform (MGESEQ-T7, Shenzhen, China). Use fastp (v0.23.0) (length_ required = 50, n_base_limit = 2) to remove low-quality reads and adaptors from the raw reads, and use Bowtie2 software (v2.3.5.1) to exclude the host reads based on the human genomes 38. Taxonomic profiling was performed using Kraken2. Quantification was calculated based on read counts. Alpha diversity was evaluated using Chao1 and Simpson indices, and beta diversity was assessed by principal component analysis based on microbial abundance profiles. Differentially enriched taxa between groups were identified using linear discriminant analysis effect size (LEfSe), and taxa with P < 0.05 and an LDA score > 2.3 were considered significantly enriched.

As for functional annotation, quality-controlled reads from each sample were *de novo* assembled into contigs using metaSPAdes (v4.0.0) with default parameters. Protein-coding sequences were subsequently predicted from the assembled contigs using Prodigal (v2.6.3), and the predicted genes were clustered using CD-HIT (v4.8.1) to construct a non-redundant gene catalog. Representative gene sequences were translated into protein sequences and subjected to functional annotation using eggNOG-mapper (v2.1.12) against the eggNOG database (v5.0.2). Functional assignments were inferred based on orthologous relationships, and corresponding functional descriptions and annotation information were obtained for the predicted proteins. To estimate gene abundance across samples, quality-controlled reads were mapped to the non-redundant gene catalog using Salmon (v1.10.3), and abundance was normalized as transcripts per million (TPM). The resulting functional annotation and abundance profiles were used for subsequent comparative analyses between experimental groups.

### 2.8 Quantification of colonic indole-3-acetic acid by the LC-ESI-MS

Colon tissues were thawed on ice, and approximately 50 mg tissue was homogenized with 1.5 mL prechilled MeOH/H_2_O (1:1, v/v). After centrifugation at 14,000 g for 10 min at 4 °C, the supernatant was collected and dried. Pellets were further extracted with CH_2_Cl_2_/MeOH (3:1, v/v), centrifuged, dried, and reconstituted in 120 μL MeOH/H_2_O (1:1, v/v) for LC-ESI-MS analysis. Indole-3-acetic acid (IAA) was measured using the Ultimate 3000 UPLC system-Orbitrap Exploris 120 mass spectrometer equipped with an electrospray ionization (ESI) source, supported by Prof Junyan Liu from Chongqing Medical University (19).

### 2.9 Cell culture and *in vitro* treatments

Caco2 cells were maintained in DMEM supplemented with 10% FBS and 1% P/S at 37°C in a humidified incubator with 5% CO_2_. The cells were seeded in 12-well and 6-well cell culture plates, cultured to 80% confluency, and then submitted to subsequent treatments. Cells were treated with sodium butyrate at 0, 1, 2, or 5 mM for 12 h. To evaluate the effect of IAA, cells were exposed to IAA (Aladdin, China) at 0, 100, 250, or 500 μM for 12 h. For PPARG activation, cells were treated with pioglitazone (MedChemExpress, China) at 20 μM for 12 h.

To assess potential cytotoxicity of sodium butyrate under the same treatment conditions, Caco-2 cells were treated with sodium butyrate at 0, 1, 2, or 5 mM for 12 h, and cell viability was subsequently assessed using a Cell Counting Kit-8 (CCK-8) according to the manufacturer’s instructions (Beyotime, China). Absorbance was measured at 450 nm using a microplate reader, and cell viability was normalized to the untreated control.

### 2.10 siRNA-mediated PPARG knockdown

PPARG knockdown in Caco-2 cells was performed using siRNA transfection. Briefly, cells were seeded in 12-well plates 24 h before transfection to reach approximately 70-80% confluence the next day. Before transfection, the culture medium was replaced with fresh complete DMEM. For each well, 40 pmol PPARG siRNA (Tsingke biology, China) or negative control siRNA was diluted in 50 μL serum- and antibiotic-free DMEM and gently mixed. Then, 1.6 μL transfection reagent (#TSV405, Tsingke biology, China) was added to the siRNA solution, mixed gently, and incubated at room temperature for 20 min to allow complex formation. The siRNA–transfection reagent mixture was then added dropwise to each well. After 12 h, the medium was replaced with fresh complete DMEM, and cells were cultured for an additional 36 h. For IAA stimulation, transfected and control cells were treated with IAA (500 μM) or vehicle for 12 h before harvest.

### 2.11 RNA extraction and quantitative real-time PCR

Total RNA was isolated from colonic tissues and cultured cells using TRIzol reagent (Vazyme, China). For reverse transcription, 1 µg of RNA from each sample was converted into cDNA with the High-Capacity cDNA Reverse Transcription Kit (Vazyme, China) following the manufacturer’s protocol. Quantitative real-time PCR was subsequently performed using SYBR Green qPCR Master Mix (Vazyme, China). *Gapdh* and *β-actin* were used as internal reference genes, and relative mRNA expression was determined by the ΔΔCt method. Primer sequences are present in **Supplementary Table 1**.

### 2.12 Western blot analysis

Caco-2 cells were lysed in RIPA buffer supplemented with protease and phosphatase inhibitors to extract total protein. Cell lysates were centrifuged at 12,000 rpm for 30 min at 4 °C, and the supernatants were collected for protein quantification using a BCA Protein Assay Kit (Beyotime, China). Equal amounts of protein (20 μg per sample) were loaded onto 10% SDS-PAGE gels and then transferred to polyvinylidene difluoride membranes (PVDF; Millipore, Billerica, MA, USA). After blocking with 5% skim milk for 1 h, membranes were incubated overnight at 4 °C with primary antibodies against CLDN2 (1:500; Proteintech, 26912-1-AP, China), PPARG (1:1,000; Proteintech, 81490-5-AP, China), and β-actin (1:3,000; #4967, Cell Signaling Technology, Danvers, MA, USA). Membranes were subsequently washed with Tris-buffered saline containing 0.01% Tween-20 and incubated with goat anti-rabbit secondary antibody (1:5,000; Cell Signaling Technology, Danvers, MA, USA) for 1 h at room temperature. Immunoreactive bands were detected using enhanced chemiluminescence reagent (ECL, Vazyme, China) and imaged with a Bio-Rad imaging system. Band intensities were quantified using ImageJ software, with β-actin used as the housekeeping control. Relative protein expression was calculated as the densitometric intensity of the target protein normalized to β-actin.

### 2.13 Statistical analysis

Statistical analyses were performed using GraphPad Prism v10. Data are expressed as mean ± SEM. For comparisons between two independent groups, an unpaired two-tailed Student’s t-test was used. Longitudinal datasets involving treatment and time were analyzed using two-way ANOVA. Associations between functional abundance and the abundance of gut microbial taxa were assessed using Pearson’s correlation coefficient for normally distributed data and Spearman’s rank correlation coefficient for non-normally distributed data. A value of P < 0.05 was considered statistically significant. Statistical significance in figures was defined as *P < 0.05, **P < 0.01, ***P < 0.001, and **P < 0.0001; ns indicates no statistical significance.

## 3 Results

### 3.1 Dietary butyrate improves colonic barrier-associated parameters and decreases plasma LPS levels

After 9 weeks of HFD feeding, as compared to controls, butyrate-treated mice had a significantly longer length of large intestine but not small intestine (**Supplementary Figure 1A**). Histological analysis of colonic sections showed that butyrate supplementation increased crypt depth and muscular layer width compared with HFD-fed controls, suggesting favorable colonic structural remodeling (**Figure 1A**). Moreover, LPS levels in both cardiac blood and portal vein blood were markedly reduced in butyrate-treated mice, consistent with reduced circulating exposure to gut-derived endotoxin (**Figure 1B**). We next assessed tight junction-associated gene expression in colonic tissues. Butyrate supplementation did not affect the mRNA levels of *Zo-1* or *Ocln*, but significantly increased *Cldn2* mRNA expression (**Figure 1C**), as well as CLDN2 protein expression, as determined by immunohistochemical staining (**Figure 1D**).

**Figure 1:**
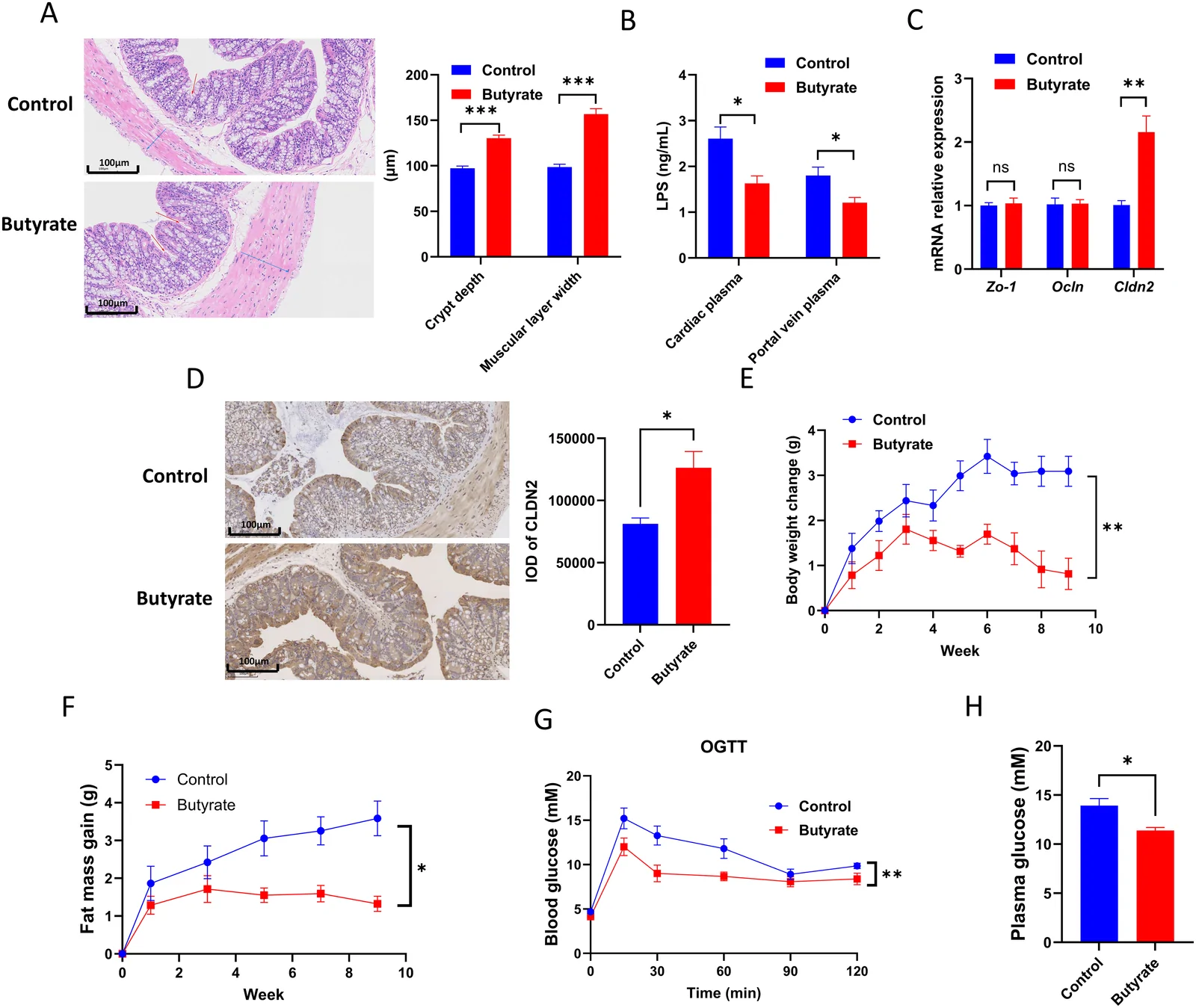
Dietary butyrate ameliorates colonic barrier integrity and decreases plasma LPS levels. Mice were fed with HFD w/o dietary butyrate (5% w/w in food). Crypt depth (red arrow) and muscular layer (blue line) width of colon were assessed after H&E staining **(A)**. Lipopolysaccharides (LPS) level was measured in cardiac plasma and portal vein plasma **(B)**. Colon mRNA level of canonical tight junction proteins was evaluated by quantitative polymerase chain reaction (qPCR) **(C)**. Integrated optical density (IOD) of colon CLDN2 (CLDN2) was analyzed by immunohistochemistry **(D)**. Weekly body weight **(E)** and fat mass change **(F)** over a 9-week period, oral glucose tolerance test (OGTT) in week 6 **(G)**, and plasma glucose level in week 8 **(H)** were determined. Data are expressed as mean ± SEM (n= 6-7). HFD, high-fat diet; ZO-1, zonula occludens-1; OCLN, occluding; SEM, standard error of the mean. Statistical significance: *p < 0.05, **p < 0.01, ***p < 0.001.

In addition, dietary butyrate supplementation significantly reduced body weight gain and fat mass gain without affecting lean mass (**Figures 1E, F**; **Supplementary Figure 1B**). Consistently, butyrate supplementation improved glucose tolerance and decreased plasma glucose levels, while plasma triglyceride and total cholesterol levels were unchanged (**Figures 1G, H**; **Supplementary Figures 1C, D**). Weight of gonadal white adipose tissue and interscapular brown adipose tissue were decreased in butyrate group (**Supplementary Figure 1E**).

Together, these data indicate that dietary butyrate induces favorable changes in multiple colonic barrier-associated parameters, including colonic tissue remodeling, reduced systemic endotoxin exposure, and altered expression of tight junction-associated components.

### 3.2 Dietary butyrate upregulates the expression of antimicrobial peptides Reg-family and PPARG in colon

To further explore the molecular basis underlying the colonic barrier-related effects of dietary butyrate, we performed RNA-seq analysis of colonic tissues from HFD-fed mice with or without butyrate supplementation. Transcriptomic profiling identified 583 differentially expressed genes, including 245 downregulated and 338 upregulated genes in the butyrate-treated group (**Figure 2A**). Among the upregulated genes, *Reg3b* and *Reg3g*, two antimicrobial peptide-related genes involved in epithelial defense, were markedly increased, suggesting that dietary butyrate may enhance antimicrobial and epithelial defense-associated responses in the colon (**Figure 2A**). To further assess pathway-level changes, we performed GSVA based on the whole transcriptomic expression matrix. The inflammatory response signature was decreased in colonic tissues from butyrate-treated mice (**Figure 2B**), which was supported by reduced colonic *Tnfa* and *Il-1b* mRNA expression in subsequent qPCR analysis (**Figure 2C**). In parallel, qPCR validation confirmed that multiple Reg-family members, including *Reg3a*, *Reg3b*, *Reg3g*, and *Reg4*, were upregulated after butyrate supplementation (**Figure 2D**). Immunohistochemical staining further showed stronger colonic REG3γ and REG4 immunoreactivity in the butyrate group, as indicated by increased Integrated optical density (IOD) values (**Figures 2E, F**). In addition to the reduced inflammatory response signature, GSVA also revealed an increased peroxisome-related signature in butyrate-treated colons (**Figure 2B**). Indeed, butyrate significantly increased *Pparg* mRNA expression (**Figures 2A, G**) and PPARγ protein expression (**Figure 2H**). Together, these findings indicate that dietary butyrate suppresses colonic inflammatory signaling while enhancing Reg-family antimicrobial peptide expression and PPARγ-related colonic responses under HFD feeding.

**Figure 2:**
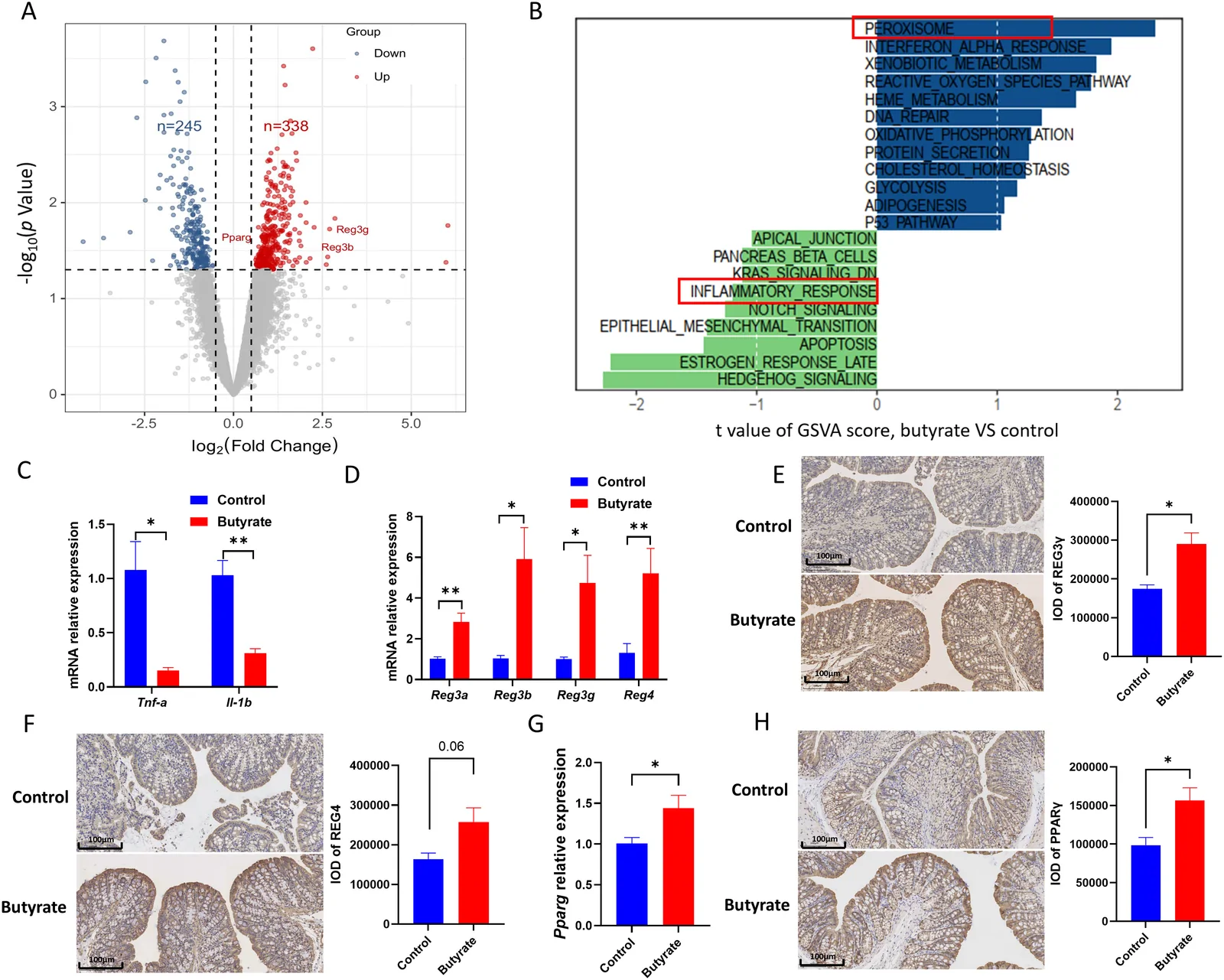
Dietary butyrate upregulates the expression of antimicrobial peptides Reg-family and PPARG in colon. RNA-seq analysis of colonic tissues from HFD-fed mice w/o dietary butyrate was performed. 245 downregulated and 338 upregulated differential expression genes were identified in RNA-seq **(A)**. Gene set variation analysis identified downregulated inflammatory response items and upregulated peroxisome items **(B)**. Colon mRNA level of canonical inflammatory markers **(C)** and antimicrobial peptides **(D)** were evaluated by quantitative polymerase chain reaction (qPCR). IOD of colon REG3γ **(E)** and REG4 **(F)** were analyzed by immunohistochemistry **(E)**. Colon mRNA level of *Pparg* was evaluated by qPCR **(G)**. IOD of colon PPARγ was analyzed by immunohistochemistry **(H)**. Data are expressed as mean ± SEM (n = 4-5). HFD, high-fat diet; *Tnf-a*, tumor necrosis factor-α; *Il-1b*, interleukin-1beta; SEM, standard error of the mean. Statistical significance: *p < 0.05, **p < 0.01.

Next, we used Caco-2 cells as a reductionist epithelial model to examine whether the *in vivo* upregulation of PPARγ, REG4, and CLDN2 could be reproduced by direct exposure to butyrate. To exclude overt cytotoxicity under the conditions used for the *in vitro* experiments, Caco-2 cell viability was assessed by CCK-8 assay. Sodium butyrate at 1–5 mM for 12 hours did not significantly affect cell viability compared with the untreated control (**Supplementary Figure 2**). In contrast to the effects observed in the colon *in vivo*, butyrate dose-dependently decreased the mRNA expression of *PPARG*, *REG4*, and *CLDN2* in Caco-2 cells (**Supplementary Figure 2B**). Consistently, butyrate decreased PPARG and CLDN2 protein levels (**Supplementary Figure 2E**). These findings indicate that the *in vivo* upregulation of PPARγ, REG4, and CLDN2 in HFD mice cannot be explained solely by a direct epithelial cell-autonomous response to butyrate and may depend on the broader intestinal context.

### 3.3 Gut microbiota contributes to the beneficial effects of dietary butyrate on colonic barrier-associated parameters

To determine whether the gut microbiota contributes to the effects of dietary butyrate under HFD feeding, we performed FMT from butyrate-treated donors into antibiotic-induced microbiota-depleted recipient mice. After 3 weeks of antibiotic treatment, fecal DNA content was reduced by more than 90% (**Figure 3A**). At 1 week after FMT, fecal DNA content was comparable between donor and recipient mice, confirming efficient recipient microbiota depletion and recolonization (**Figure 3B**). During the subsequent 15-week HFD challenge, recipients of microbiota from butyrate-treated donors showed reduced body weight gain (**Figure 3C**) and fat mass gain (**Figure 3D**), with no change in lean mass (**Supplementary Figure 3A**). These mice also exhibited improved glucose tolerance (**Figure 3E**), lower plasma glucose levels (**Figure 3F**), without significantly altered plasma triglyceride levels (**Supplementary Figure 3B**) and total cholesterol levels (**Supplementary Figure 3C**). After sacrifice, weight of gonadal white adipose tissue and interscapular brown adipose tissue decreased in butyrate recipient group (**Supplementary Figure 3D**). These findings demonstrate that microbiota shaped by dietary butyrate can partially transfer the associated metabolic phenotype under HFD feeding. 

**Figure 3:**
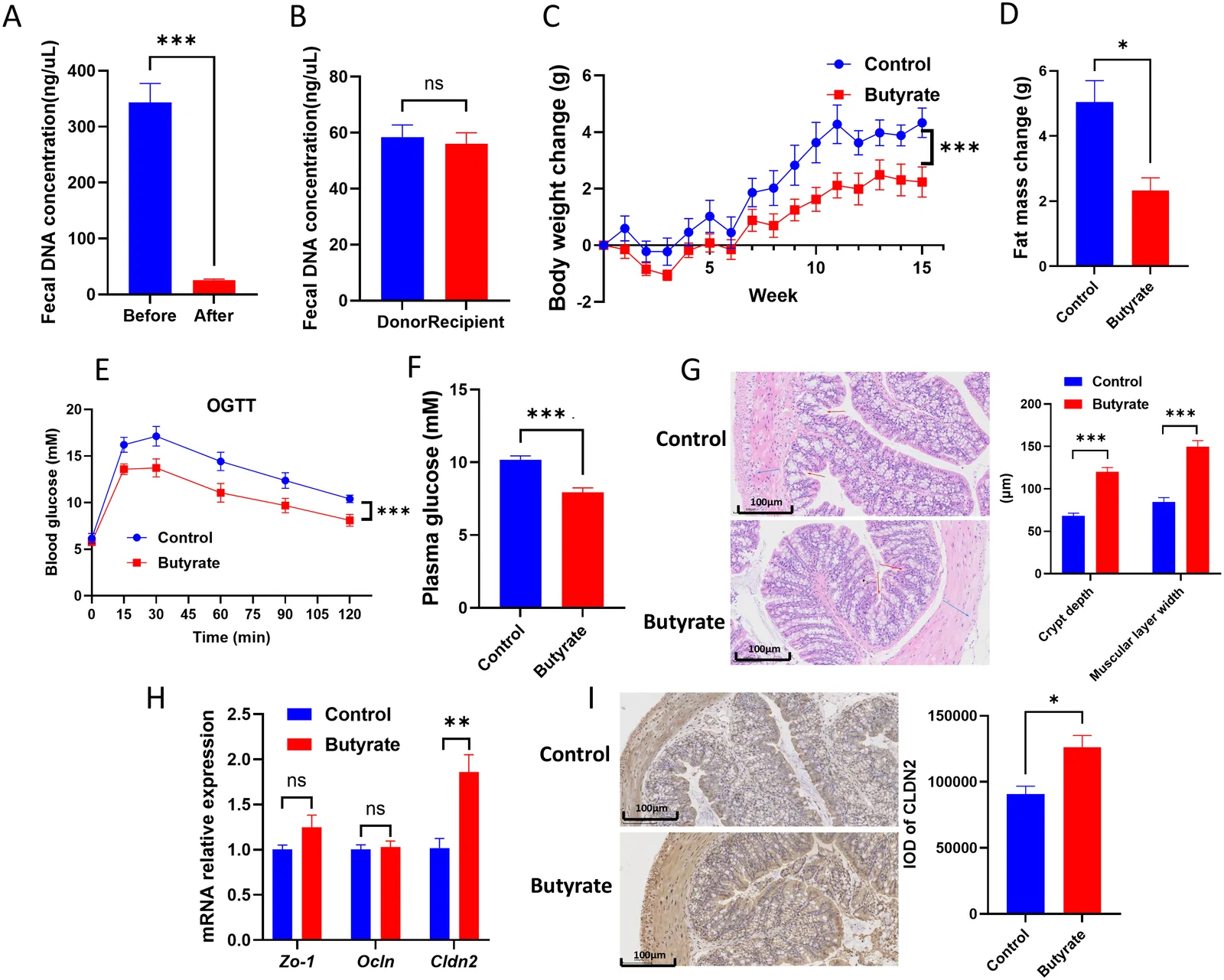
Gut microbiota contributes to the beneficial effects of dietary butyrate on colonic barrier-associated parameters. Fecal content of HFD-fed donor mice w/o dietary butyrate were transplanted to HFD-fed recipient mice. The fecal DNA contents in recipient mice before and after antibiotics-induced microbiota depletion (AIMD) **(A)**, as well as the fecal DNA contents in donor and recipient mice after fecal microbiota transplantation (FMT) **(B)** were determined. Weekly body weight change **(C)** and fat mass change **(D)** over a 15-week period, oral glucose tolerance test (OGTT) in week 9 **(E)**, plasma glucose levels of recipient mice in week 13 **(F)** were determined. Crypt depth (red arrow) and muscular layer (blue line) width of colon of recipient mice were assessed after H&E staining **(G)**. Colon mRNA levels of canonical tight junction proteins were evaluated by qPCR **(H)**. Integrated optical density (IOD) of CLDN2 of recipient mice was analyzed by immunohistochemistry **(I)**. Data are expressed as mean ± SEM (n = 6-8). HFD, high-fat diet; ZO-1, zonula occludens-1; OCLN, occluding; SEM, standard error of the mean. Statistical significance: *p < 0.05, **p < 0.01, ***p < 0.001.

We next examined whether the transplanted microbiota recapitulated the colonic barrier-related changes observed after dietary butyrate supplementation. As compared with control-recipient mice, histological analysis showed that butyrate-recipient mice exhibited increased crypt depth and muscular layer width (**Figure 3G**). Consistently, qPCR analysis showed that both mRNA levels of *CLDN2* expression (**Figure 3H**) and CLDN2 protein expression (**Figure 3I**) were significantly increased in butyrate-recipient mice. In parallel, butyrate-recipient mice showed decreased colonic mRNA expression of *TNF-a* and *IL-1b* (**Figure 4A**), indicating attenuated colonic inflammatory responses. Moreover, the expression of epithelial defense- and repair-associated factors, including *Reg3a*, *Reg3b*, *Reg3g*, and *Reg4*, was significantly upregulated in the colon of butyrate-recipient mice (**Figure 4B**). Immunohistochemical staining further showed stronger REG3γ and REG4 signals in butyrate-recipient mice than in control-recipient mice (**Figures 4C, D**). In addition, *Pparg* mRNA expression was increased in colonic tissues from butyrate-recipient mice, accompanied by enhanced colonic PPARγ immunoreactivity (**Figures 4E, F**). Together, these findings indicate that butyrate-shaped gut microbiota was sufficient to transfer several colonic barrier-associated and anti-inflammatory features under HFD feeding, including favorable colonic structural changes, increased Reg-family and PPARγ expression, and reduced inflammatory cytokine expression.

**Figure 4:**
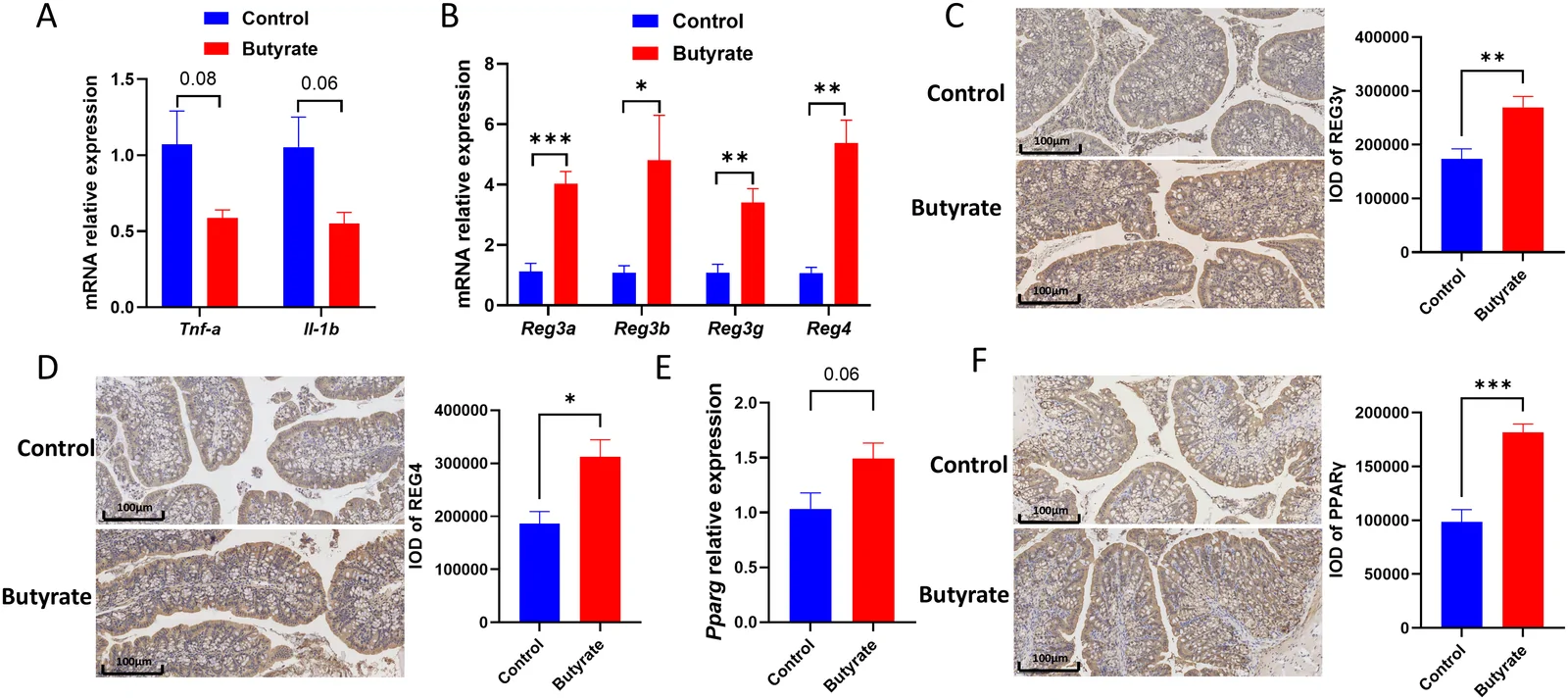
Fecal microbiota transplantation (FMT) from butyrate-treated donors upregulates antimicrobial peptides and PPARG expression in colon of recipient mice. Colon mRNA levels of canonical inflammatory markers **(A)**, antimicrobial peptides **(B)** of recipient mice were evaluated by qPCR. IOD of colon REG3γ **(C)** and REG4 **(D)** were analyzed in recipient colons by immunohistochemistry. Colon mRNA level of *Pparg* was evaluated by qPCR **(E)**. IOD of colon PPARG was analyzed by immunohistochemistry **(F)**. Data are expressed as mean ± SEM (n = 4-5). HFD, high-fat diet; *Tnf-a*, tumor necrosis factor-α; *Il-1b*, interleukin-1beta; SEM, standard error of the mean. Statistical significance: *p < 0.05, **p < 0.01, ***p < 0.001.

### 3.4 Butyrate enriches Bacteroides associated with increased microbial IAA biosynthetic potential and colonic IAA levels

To further define the microbial features underlying the transmissible effects of dietary butyrate, we performed metagenomic analysis of cecal contents from donor and recipient mice. The Chao1 and Simpson indices were not significantly different between butyrate-treated and control donor mice or between butyrate-recipient and control-recipient mice (**Figures 5A, B**). Principal component analysis also showed no clear separation among control donors, butyrate-treated donors, control recipients, and butyrate recipients (**Figure 5C**). These data indicate that dietary butyrate did not broadly reshape gut microbial α- or β-diversity in the present experimental setting, and also support the overall comparability and quality of the FMT procedure.

**Figure 5:**
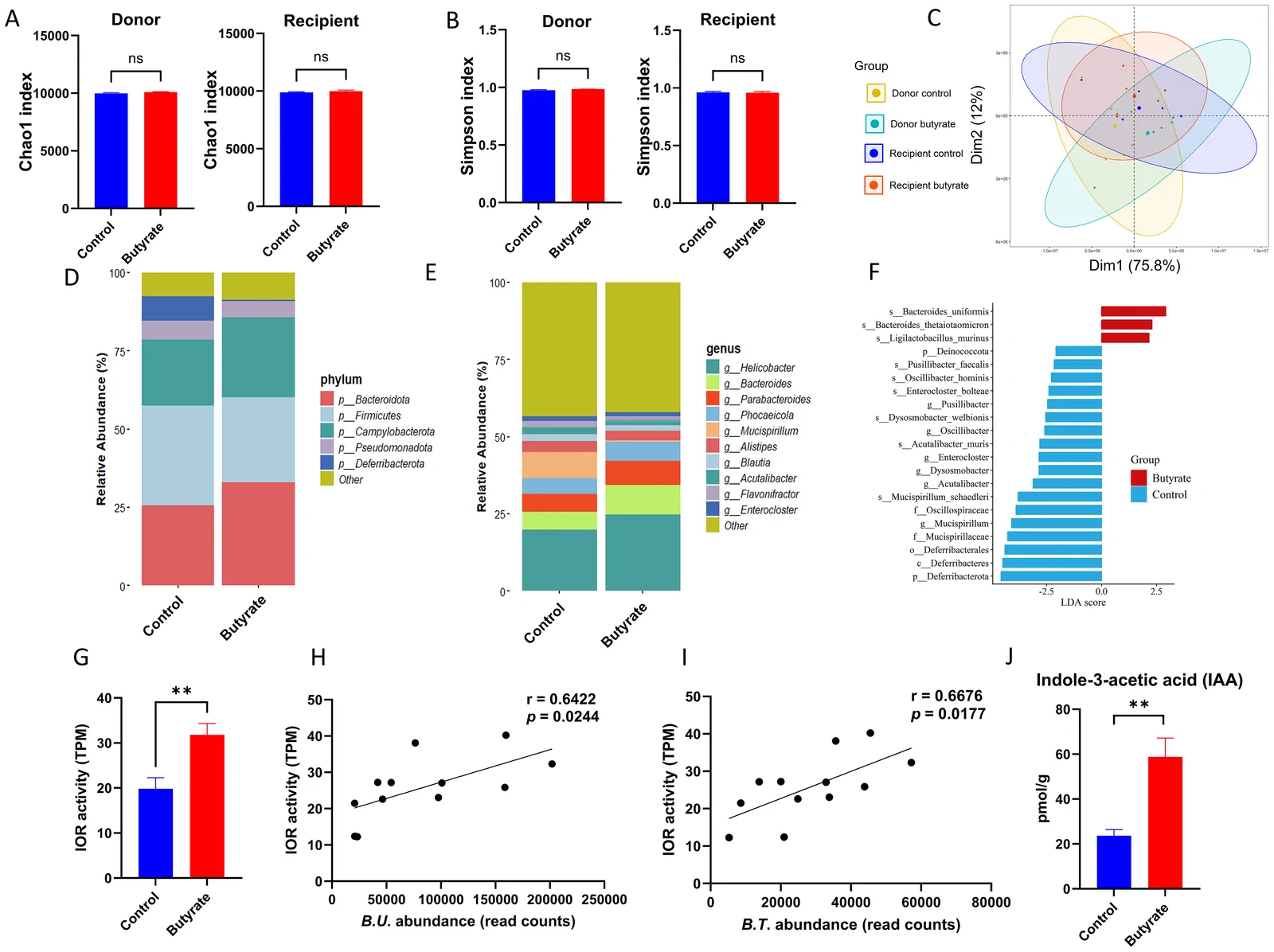
Butyrate enriches Bacteroides associated with increased microbial IAA biosynthetic potential and colonic IAA levels. α-diversity of gut bacteria of donor and recipient mice in control and butyrate group was determined, as shown in Chao1 index **(A)** and Simpson index **(B)**. β-diversity of gut bacteria of donor and recipient mice in control and butyrate group, analyzed with principle component analysis **(C)**. Composition of gut bacteria of recipient mice in control and butyrate group in phylum **(D)** and genus **(E)** level. Gut microbial taxa with over 2.3 liner discriminant analysis (LDA) score screened in recipient mice in control and butyrate group **(F)**. The abundance of indolepyruvate:ferredoxin oxidoreductase (IOR) activity was quantified based on eggNOG functional annotation and expressed as transcripts per million (TPM) **(G)**. Pearson correlations between IOR activity and the abundance of *Bacteroides uniformis* **(H)** and *Bacteroides thetaiotaomicron* **(I)** were analyzed. Colonic IAA level was determined by LC-MS **(J)**. Data are expressed as mean ± SEM (n = 4-6). HFD, high-fat diet; SEM, standard error of the mean. Statistical significance: **p < 0.01.

Despite the absence of major global diversity shifts, butyrate-recipient mice displayed distinct taxonomic remodeling. At the phylum level, *Bacteroidota* abundance was increased in butyrate-recipient mice compared with control-recipient mice (**Figure 5D**). Consistently, at the genus level, Bacteroides was enriched in the butyrate-recipient group (**Figure 5E**). Since Bacteroides has been recognized as an important microbial source of tryptophan-derived indole metabolites (20), we further examined species-level microbial signatures in recipient mice. Linear discriminant analysis revealed that *Bacteroides uniformis* and *Bacteroides thetaiotaomicron* were the top-enriched species in butyrate-recipient mice compared with control-recipient mice (**Figure 5F**). Notably, both species have previously been linked to microbial IAA metabolism (21, 22), suggesting that their enrichment may be associated with increased microbial IAA biosynthetic potential.

Functional annotation of the metagenomic data revealed an increase in indolepyruvate ferredoxin oxidoreductase (IOR) activity in butyrate-recipient mice compared with control-recipient mice (**Figure 5G**). Notably, IOR catalyzes the conversion of indolepyruvate to indole-3-acetyl-CoA, an intermediate involved in microbial IAA biosynthesis (**Figure 4**), linking this functional alteration to the observed enrichment of IAA-producing *Bacteroides*. Consistently, IOR activity was positively correlated with the abundance of *B. uniformis* and *B. thetaiotaomicron* in recipient mice (**Figures 5H, I**). To determine whether these taxonomic and functional changes were accompanied by increased IAA in the colon, we performed quantitative LC/MS analysis of colonic tissues from HFD-fed mice treated with or without sodium butyrate gavage at the same dose used in the dietary butyrate experiment. Butyrate treatment significantly elevated colonic IAA levels compared with controls (**Figure 5J**). Collectively, these findings indicate that sodium butyrate promotes *Bacteroides*-associated taxonomic remodeling accompanied by increased IOR activity and enhanced colonic IAA production.

### 3.5 IAA increases *REG4* and CLDN2 expression through PPARG-dependent signaling in Caco-2 cells

To further determine the direct effects of IAA on colonic cells, we again used Caco-2 cells as an *in vitro* model. IAA increased the mRNA expression of *PPARG*, *CLDN2*, and *REG4* in a dose-dependent manner (**Figures 6A–C**). Consistently, western blot analysis showed that IAA also increased the protein expression of CLDN2 and PPARG (**Figures 6D–F**).

**Figure 6:**
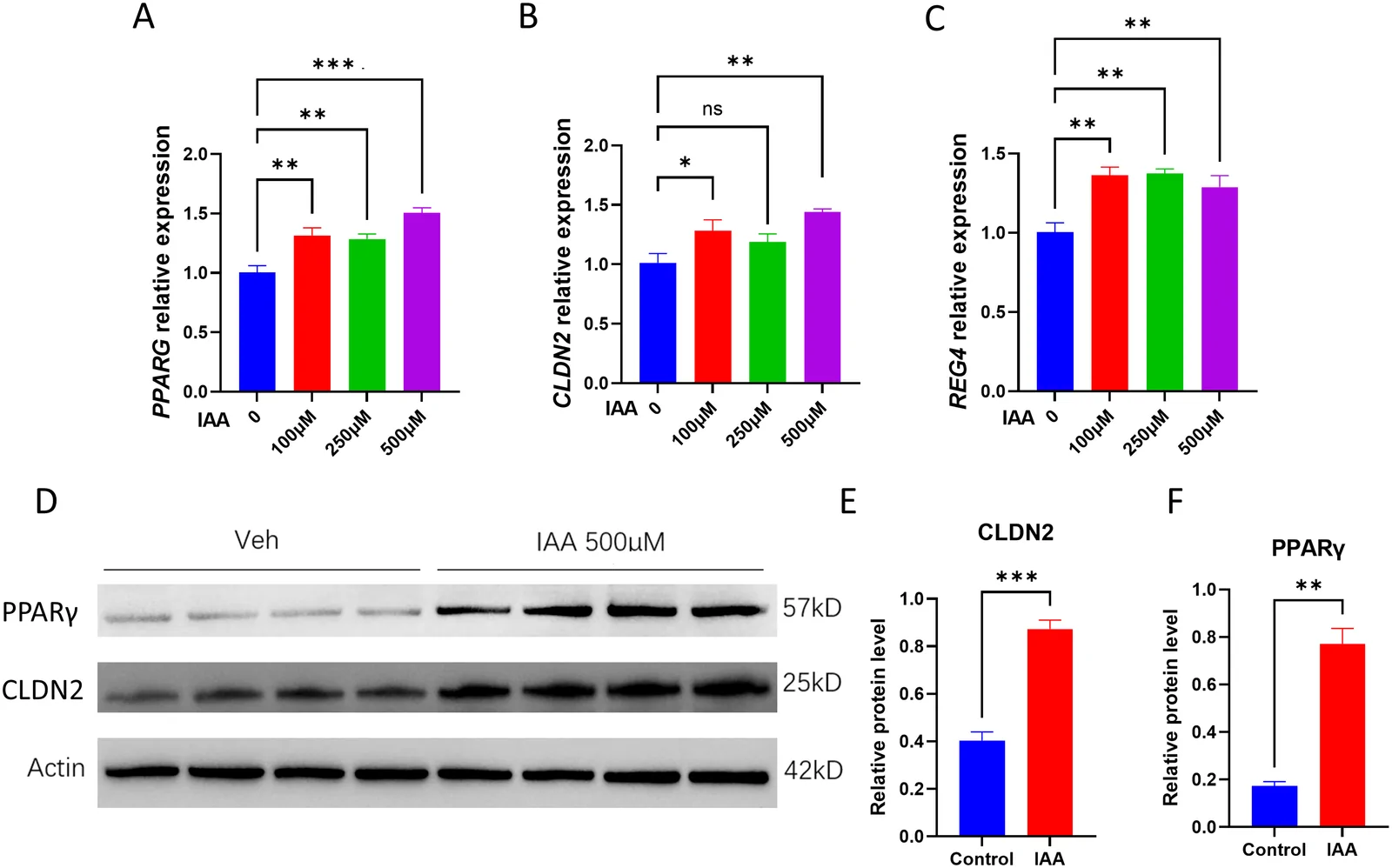
IAA increases PPARG, *REG4* and CLDN2 expression. The mRNA expression of peroxisome proliferator-activated receptor gamma (*PPARG*) **(A)**, *Claudin2* (*CLDN2*) **(B)**, and *REG4* **(C)**, in a dose-dependent manner. IAA increased protein expression of CLDN2 and PPARG **(D-F)**. Data are expressed as mean ± SEM (n = 4). SEM, standard error of the mean. Statistical significance: *p < 0.05, **p < 0.01, ***p < 0.001.

Moreover, treatment of Caco-2 cells with the PPARG agonist pioglitazone significantly increased the mRNA expression of *REG4* (**Figure 7A**) and *CLDN2* (**Figure 7B**). In agreement with these transcriptional changes, pioglitazone also increased CLDN2 protein expression, as shown by western blot analysis and quantitative protein assessment (**Figures 7C, D**). These findings thus reveal that PPARG activation promotes *REG4* and CLDN2 expression in Caco-2 cells. To examine whether PPARG is required for the IAA-induced upregulation of *REG4* and CLDN2, we next we knocked down *PPARG* expression using siRNA. While IAA increased *PPARG* mRNA expression, *PPARG* knockdown markedly reduced *PPARG* expression in both the absence and presence of IAA (**Figure 7E**). Importantly, *PPARG* knockdown neutralized the IAA-induced increase in *REG4* mRNA expression (**Figure 7F**). Similarly, IAA-induced *CLDN2* mRNA upregulation was markedly attenuated after *PPARG* knockdown, with *CLDN2* expression in the IAA plus siPPARG group being significantly lower than that in the IAA-treated group (**Figure 7G**). At the protein level, western blot analysis confirmed that IAA increased PPARG and CLDN2 protein expression, whereas *PPARG* knockdown suppressed PPARG expression and abolished the IAA-induced increase in CLDN2 protein expression (**Figures 7H–J**). Together, these results support a PPARG-dependent role in the IAA-induced upregulation of *REG4* and CLDN2 in Caco-2 cells.

**Figure 7:**
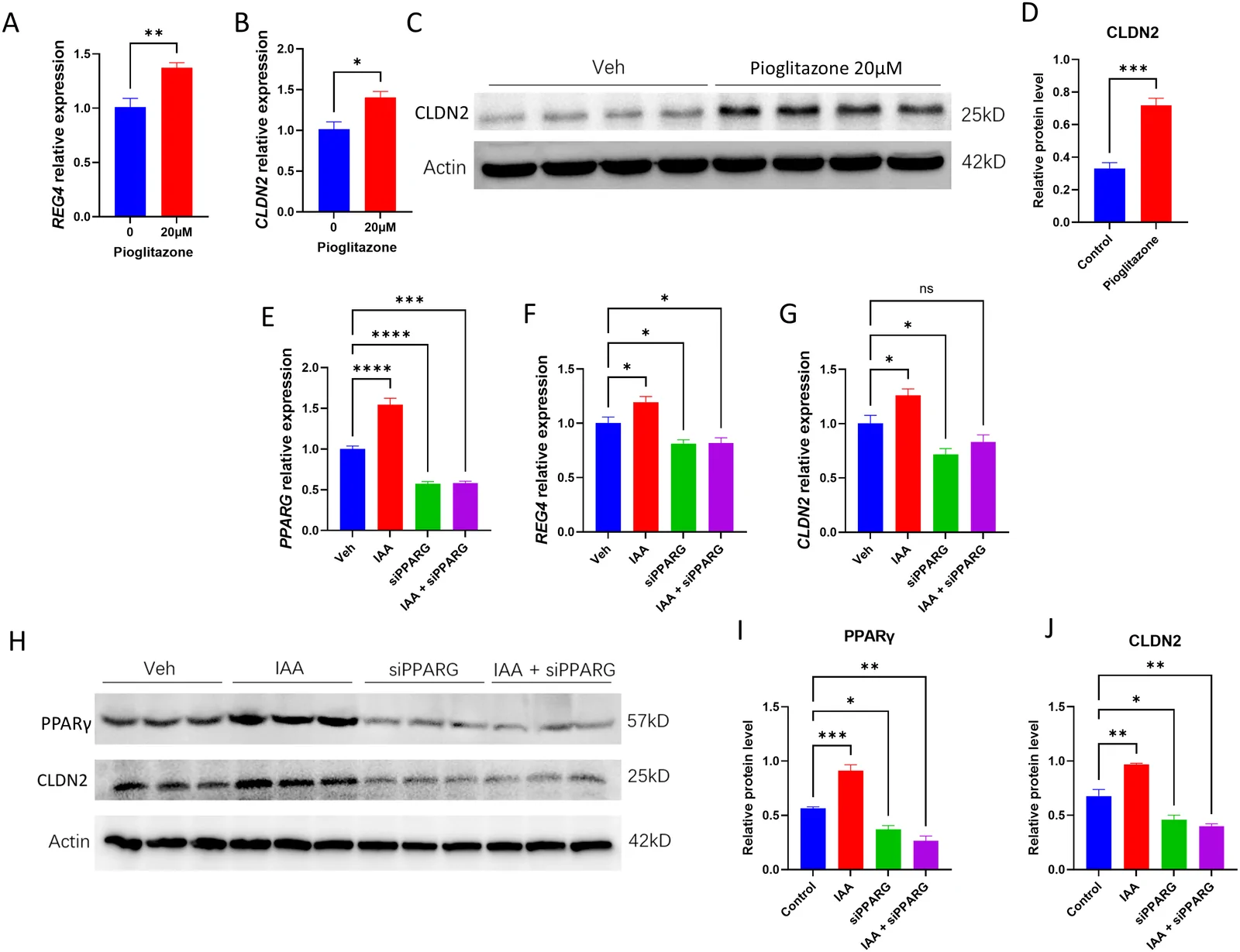
IAA increases REG4 and CLDN2 expression through PPARG-dependent signaling. Pioglitazone, a PPARG agonist, increased mRNA expression of *REG4* **(A)** and *CLDN2* **(B)**. Pioglitazone increased protein expression of CLDN2 **(C, D)**. Knocking down PPARG with siRNA neutralized the increase in mRNA expression of *REG4* and *CLDN2* **(E-G)**, and protein expression of CLDN2 **(H-J)**. Data are expressed as mean ± SEM (n = 3-4). SEM, standard error of the mean. Statistical significance: *p < 0.05, **p < 0.01, ***p < 0.001, ****p < 0.0001.

## 4 Discussion

In the present study, dietary butyrate improved multiple aspects of colonic mucosal homeostasis under HFD feeding. FMT experiments further showed that dietary butyrate-shaped gut microbiota could transfer several metabolic and colonic phenotypes. Butyrate-associated microbial remodeling was characterized by enrichment of Bacteroides-related taxa and increased microbial IAA biosynthetic potential, accompanied by increased colonic IAA levels. In Caco-2 cells, IAA induced *REG4* and CLDN2 expression in a PPARG-dependent manner. Together, these findings suggest a potential mechanistic framework linking butyrate-associated microbial remodeling, microbial IAA metabolism, and epithelial PPARG signaling (as shown in **Figure 8**).

**Figure 8:**
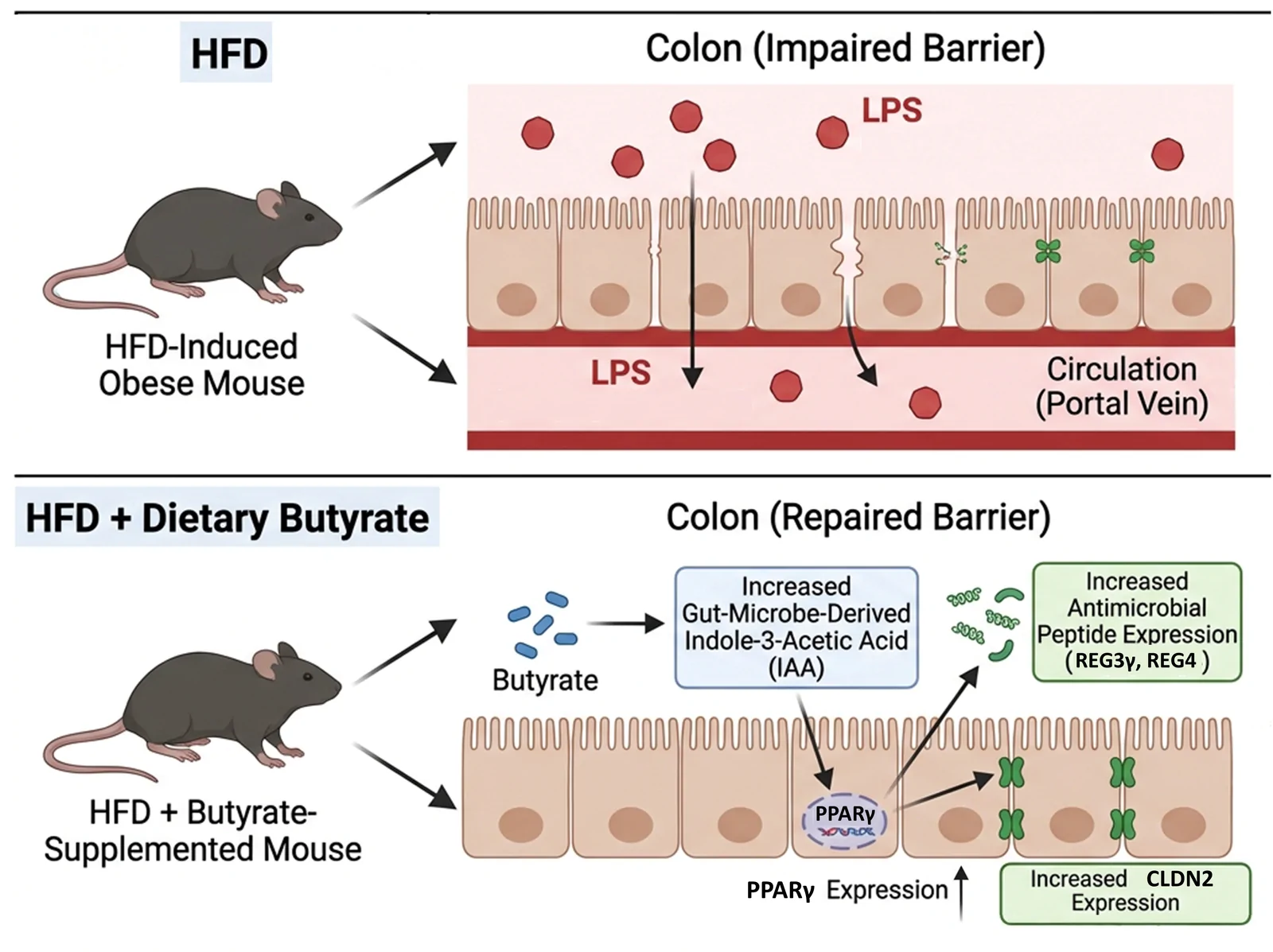
Proposed conceptual model integrating the microbial, metabolic, and epithelial findings of the present study. Dietary butyrate-associated gut microbial remodeling and increased colonic indole-3-acetic acid (IAA) may contribute to PPARγ-related epithelial responses and improved colonic mucosal homeostasis under HFD feeding. CLDN2, Claudin2.

HFD-induced metabolic dysfunction is closely linked to impaired intestinal barrier integrity and metabolic endotoxemia. Increased gut-derived LPS exposure can activate inflammatory signaling and thereby contribute to obesity, insulin resistance, and chronic low-grade inflammation (4). In the present study, dietary butyrate reduced body weight gain and fat mass accumulation and improved glucose tolerance in HFD-fed mice, consistent with previous studies showing that butyrate alleviates diet-induced metabolic abnormalities (23, 24). Importantly, butyrate reduced LPS levels in both portal vein blood and systemic circulation, consistent with reduced circulating exposure to gut-derived endotoxin. This was accompanied by reduced colonic *Tnfa* and *IL1b* expression, suggesting attenuation of local inflammatory responses. These findings are in line with previous reports that butyrate improves HFD-induced intestinal barrier disruption and reduces endotoxin-related inflammatory alterations (25, 26). Taken together, the metabolic benefits of dietary butyrate were accompanied by favorable changes in multiple colonic barrier-associated parameters and reduced colonic inflammatory burden.

In our study, butyrate increased colonic *CLDN2* expression without significantly altering *Zo-1* or *Ocln* expression. This finding differs from a recent study in HFD-fed (40% kcal derived from fat) prediabetic mice, in which butyrate reduced colonic *CLDN2* expression while reinforcing tight junction-mediated epithelial barrier function (27). This discrepancy may be related to differences in dietary fat burden and disease severity. The higher-fat challenge (60% kcal derived from fat) used in our model may impose stronger epithelial stress, inflammatory pressure, and tight junction remodeling, as HFD has been shown to induce epithelial ER/oxidative stress, inflammatory cytokine expression, mucosal barrier impairment, tight junction restructuring, and endotoxin leakage (28, 29). CLDN2 is an integral component of the tight junction complex, but its functional implication may vary depending on the pathological context. Therefore, increased CLDN2 expression in our model may reflect context-dependent reorganization of tight junction composition during butyrate-mediated barrier adaptation under aggravated HFD-induced mucosal stress, rather than a simple change in canonical sealing junction markers.

Beyond tight junction-associated changes, butyrate also increased colonic Reg-family and PPARγ expression. Reg3-family proteins are microbiota-responsive epithelial defense factors (30, 31), while epithelial *Pparg* mRNA contributes to intestinal anti-inflammatory protection (32, 33). These changes, together with reduced LPS translocation, decreased inflammatory cytokine expression, and improved colonic architecture, suggest that dietary butyrate enhances colonic barrier-related homeostasis through coordinated effects on endotoxin leakage, inflammatory tone, epithelial defense factors, and tight junction composition under HFD feeding.

Importantly, both recipient groups underwent the same antibiotic pretreatment before FMT, thereby minimizing differences attributable to antibiotic exposure between the two recipient groups. Nevertheless, the present FMT design demonstrates microbial contribution and transferability rather than establishing that gut microbiota is strictly required for the effects of dietary butyrate. Although microbiota depletion during butyrate treatment could directly test such necessity, interpretation of intestinal phenotypes in this setting may be complicated by the effects of antibiotics themselves on the intestinal barrier. Recent evidence shows that commonly used microbiota-depleting antibiotics, including ampicillin, metronidazole, neomycin, and vancomycin, can disrupt the colonic mucus barrier, with some effects occurring independently of microbiota alterations (34). Consistent with a relevant role of gut microbiota, our previous study using the same APOE*3-Leiden.CETP model demonstrated an important microbiota-dependent component of the metabolic benefits of dietary butyrate (13).

The present FMT experiment provides functional evidence that gut microbiota shaped by dietary butyrate contributes to and can transfer several of the observed colonic phenotypes. Microbiota from butyrate-treated donors recapitulated multiple colonic barrier-associated features in HFD-fed recipients, including favorable colonic structural changes, reduced *Tnf-α* and *Il-1b* expression, and increased Reg-family, PPARγ, and CLDN2 expression. Given the fact that butyrate decreased *Pparg, Reg4*, and *CLDN2* expression in Caco-2 cells, contrasting with upregulation of these genes in the colon *in vivo*, the colonic response to dietary butyrate is unlikely to be driven by a direct epithelial cell-autonomous effect. Rather, dietary butyrate may modulate the intestinal microenvironment, including microbiota-associated signals, thereby shaping the observed colonic responses under HFD feeding. Therefore, our findings extend previous observations that dietary butyrate modulates gut microbiota under HFD feeding (35), FMT can partially transfer microbiota-dependent host phenotypes (36, 37), and gut microbiota–intestinal barrier interactions are closely associated with HFD-induced metabolic disease (38). Nevertheless, these results do not exclude direct host effects of butyrate, and the effects of dietary butyrate on colonic homeostasis may involve both direct host signaling and indirect microbiota-derived pathways (9, 11).

Although butyrate did not markedly alter overall microbial α- or β-diversity, it selectively enriched *Bacteroidota* and *Bacteroides* in recipient mice, with *Bacteroides uniformis* and *Bacteroides thetaiotaomicron* emerging as the top-enriched species. This pattern may reflect butyrate-mediated remodeling of the intestinal ecological niche rather than direct nutritional selection by butyrate. As a major microbial fermentation product and energy source for colonocytes, butyrate supports epithelial metabolism, barrier integrity, mucus-related homeostasis, and a stable anaerobic luminal environment (39), which may favor distal gut-adapted anaerobic commensals such as *Bacteroides*. These taxa possess extensive glycan-utilization systems and can exploit both dietary and host-derived glycans (40, 41). *B. uniformis* has been reported to ameliorate metabolic and immunological dysfunction in HFD-induced obese mice (42, 43), while *B. thetaiotaomicron* can modulate tryptophan metabolism and AhR-related immune responses during colonic inflammation (44). Importantly, both species have been linked to IAA-related microbial metabolism: *B. uniformis IM01* increases colonic and systemic IAA levels (21), and *B. thetaiotaomicron* have the capacity to produce IAA dependent on pH and carbohydrate availability (22). In the present study, functional metagenomic analysis further showed increased IOR-related functional abundance in butyrate-recipient mice, which positively correlated with the abundance of both *B. uniformis* and *B. thetaiotaomicron*. As IOR participates in microbial IAA biosynthesis (45, 46), these findings strengthen the association between butyrate-induced *Bacteroides* enrichment and increased microbial IAA biosynthetic potential. Together with the elevated colonic IAA levels observed following butyrate treatment, these data support the possibility that dietary butyrate promotes an IAA-associated microbial metabolic configuration. Recent work in experimental liver fibrosis has shown that alterations in butyrate, can be coupled to host signaling responses involving the Wnt/β-catenin pathway (47). Our findings extend this metabolite–host signaling concept by suggesting that dietary butyrate may additionally reshape the intestinal microbial-metabolic environment, accompanied by increased IAA availability and a distinct PPARγ-related epithelial response. Previous studies have mainly linked microbial indole metabolites to epithelial homeostasis through AhR- or PXR-dependent mechanisms (15, 48), our findings suggest an additional epithelial PPARγ-related pathway. Given that epithelial PPARγ regulates intestinal inflammation and mucosal homeostasis (49, 50), and can protect barrier integrity through tight-junction-dependent mechanisms (51), our findings support a PPARγ-associated epithelial response to IAA that may link microbial indole metabolism with barrier-related gene expression.

By combining IAA stimulation, pioglitazone-mediated PPARγ activation, and PPARγ knockdown, our findings support a PPARG-dependent epithelial response underlying IAA-induced REG4 and CLDN2 expression in Caco-2 cells. This directional evidence links microbial IAA to barrier-related epithelial gene regulation and provides the basis for discussing the biological relevance of *REG4* and CLDN2 as downstream effectors. The downstream molecules identified in this pathway further support their biological relevance. REG4 has been implicated in colonic epithelial niche organization, epithelial repair, and antibacterial defense (52, 53). Therefore, IAA-induced *REG4* expression may reflect activation of an epithelial defense- or repair-associated program. As for CLDN2, it is often regarded as a pore-forming tight junction protein associated with increased epithelial permeability during intestinal inflammation (54). CLDN2 also has context-dependent functions beyond passive leak formation, including roles in epithelial remodeling and ion-water homeostasis (55). In our study, CLDN2 upregulation occurred together with reduced LPS translocation, decreased inflammatory cytokine expression, increased Reg-family expression, and improved colonic architecture. Therefore, IAA–PPARγ-induced *REG4* and CLDN2 expression may represent a broader epithelial remodeling response that links microbial IAA signaling to barrier-related adaptation under HFD-induced stress.

The present findings may have several translational implications. First, dietary butyrate, or nutritional strategies that enhance endogenous butyrate production, could serve as a potential approach to counteract HFD-associated gut barrier impairment. This is consistent with the broader concept that short-chain fatty acids, particularly butyrate, contribute to intestinal barrier maintenance and anti-inflammatory regulation. Second, our data suggest that barrier protection may not depend solely on butyrate *per se*, but may also involve downstream microbiota-derived metabolites such as IAA. This raises the possibility that future interventions could be designed to promote IAA-producing microbial configurations or directly enhance colonic IAA availability. Recent studies have increasingly recognized IAA as a bioactive microbial metabolite with potential relevance to intestinal inflammation and barrier homeostasis (56, 57). Third, the identification of epithelial PPARγ signaling as a downstream response provides a druggable host pathway that may be leveraged for barrier repair. PPARγ is highly expressed in the colon and has been implicated in the control of intestinal inflammation, while PPARγ agonists have shown barrier-protective and anti-inflammatory effects in epithelial and experimental inflammatory settings (33, 58). Therefore, the relationship among dietary butyrate, gut microbial metabolism, IAA, and epithelial PPARγ signaling may provide a conceptual framework for future diet-, microbiota-, metabolite-, or host-targeted studies.

Several aspects of this study warrant further investigation. First, intestinal permeability was not directly assessed using functional approaches such as FITC-dextran permeability or Ussing chamber analysis. Therefore, our conclusions regarding colonic mucosal homeostasis are based on convergent barrier-associated parameters, including colonic histology, circulating LPS levels, inflammatory responses, and epithelial barrier-associated molecules, rather than a direct measurement of epithelial permeability. Future studies incorporating functional permeability assays will be needed to further validate these observations. Second, our metagenomic and metabolomic data revealed enrichment of *B. uniformis* and *B. thetaiotaomicron* together with increased colonic IAA levels, supporting a potential microbial source of IAA under dietary butyrate intervention. Future studies using mono-colonization, defined microbial consortia, or isotope-labeled tryptophan tracing would further clarify the relative contribution of these taxa to IAA production *in vivo*. Third, although our FMT and *in vitro* experiments support the involvement of IAA-related epithelial signaling in butyrate-mediated barrier protection, additional *in vivo* approaches, such as IAA supplementation or modulation of IAA-responsive host pathways, would help strengthen the causal relationship. Fourth, Caco-2 cells were used as an intestinal epithelial-like model to explore the IAA–PPARγ–*REG4*/CLDN2 signaling relationship. In addition, although sodium butyrate did not affect Caco-2 cell viability under the conditions tested, the effects of exposure duration and cellular differentiation state were not systematically examined and may influence epithelial responses to butyrate. Given the differences between Caco-2 cells and physiological mouse colonic epithelium, future validation in primary colonic epithelial cells, intestinal organoids, or (colon) epithelium-specific *Pparg*-deficient mice would provide more physiological evidence.

## 5 Conclusion

In conclusion, dietary butyrate alleviated HFD-induced metabolic dysfunction and improved multiple aspects of colonic mucosal homeostasis. Several of these phenotypic changes were partially transferable by microbiota from butyrate-treated donors and were accompanied by enrichment of Bacteroides-related taxa, increased microbial IAA biosynthetic potential, and elevated colonic IAA levels. In Caco-2 cells, IAA promoted *REG4* and CLDN2 expression in a PPARG-dependent manner. Collectively, these findings suggest a potential mechanistic framework linking butyrate-associated microbial remodeling and IAA metabolism with epithelial PPARγ responses, while further *in vivo* studies are required to establish the causal relationships among these components.

## Data Availability

The datasets presented in this study can be found in online repositories. The names of the repository/repositories and accession number(s) can be found in the article/**Supplementary Material**.
