# Supplementary material for: Dietary butyrate alleviates high-fat diet-induced colonic mucosal impairment accompanied by increased gut-derived indole-3-acetic acid and enhanced PPARγ signaling
Source: Front Endocrinol (Lausanne). 2026 Oct 21;17:1971187. doi: 10.3389/fendo.2026.1971187 (PMC13730826; doi:10.3389/fendo.2026.1971187)
Supplement: Supplementary file 1 [file SupplementaryFile1.docx]

**Supplementary Material**

SFig.1 Oral administration of sodium butyrate shortened the length of large intestine but not small intestine (A). Oral administration of sodium butyrate possessed no effect on lean mass (B). Plasma triglycerides (TG) (C) and total cholesterol (TC) (D) tested with tail vein blood in week 8. Weight of gonadal white adipose tissue (gWAT) and interscapular brown adipose tissue (iBAT) after sacrifice (E). Data are expressed as mean ± SEM (n = 6-7). HFD, high-fat diet; SEM, standard error of the mean.

SFig.2 Caco-2 cells were treated with sodium butyrate at 0, 1, 2, or 5 mM for 12 h, and cell viability was assessed using the CCK-8 assay (A). The relative mRNA expression of *Pparg* (B), *Reg4* (C), and *Cldn2* (D) was determined by quantitative real-time PCR. Representative western blots of PPARγ and CLDN2 in Caco-2 cells treated with vehicle or 5 mM sodium butyrate are shown in (E), with β-actin used as the loading control. Quantification of PPARγ (F) and CLDN2 (G) protein levels was normalized to β-actin. Data are expressed as mean ± SEM (n = 4). CCK-8, Cell Counting Kit-8; SEM, standard error of the mean.

SFig.3 FMT from sodium butyrate treatment possessed no effect on lean mass (A) in mice following HFD intervention. Plasma triglycerides (TG) (B), total cholesterol (TC) (C) of recipient mice tested with tail vein blood in week 13. Weight of gonadal white adipose tissue (gWAT) and interscapular brown adipose tissue (iBAT) of recipient mice after sacrifice (D). Data are expressed as mean ± SEM (n = 6-8). HFD, high-fat diet; SEM, standard error of the mean.

SFig.4 Schematic representation of the IOR-associated route contributing to microbial IAA biosynthesis. L-Tryptophan can be metabolized to indole-3-pyruvate (IPA). Indolepyruvate:ferredoxin oxidoreductase (IOR) catalyzes the conversion of IPA to indole-3-acetyl-CoA, an intermediate that may contribute to microbial indole-3-acetic acid (IAA) biosynthesis.

Supplementary Table 1

SUPPLEMENTARY TABLE 1 Primers involved in this study.

| **Species** | **Gene** | **Forward sequence (5’ to 3’)** | **Reverse sequence (5’ to 3’)** |
| --- | --- | --- | --- |
| Mouse | *β-Actin* | AACCGTGAAAAGATGACCCAGAT | CACAGCCTGGATGGCTACGTA |
| Mouse | *Cldn2* | ATGTGGGTGTGGAGGTTTTCT | CAGTGTCTCTGGCAAGCTGA |
| Mouse | *Gapdh* | GGGGCTGGCATTGCTCTCAA | TTGCTCAGTGTCCTTGCTGGGG |
| Mouse | *Il-1b* | TGCAAGTGTCTGAAGCAGCTATG | TAGCCACTGAAGGAGATGAGTTG |
| Mouse | *Ocln* | CTCCTCCAATGGCAAAGTGAATG | AAGTCATCCACACTCAAGGTCAG |
| Mouse | *Pparg* | ACACCCAGCTCCCATCTTTATTT | GTCAGCGGGAAGGACTTTATGTA |
| Mouse | *Tnf-a* | AGGCTGCCCCGACTACGT | GACTTTCTCCTGGTATGAGATAGCAAA |
| Mouse | *Reg3a* | ATTCTTAGTGGAGGTGAGGCTTC | CGGTTGACAGTAGAGGAAGGATC |
| Mouse | *Reg3b* | CCATGGTGAAGAGAACAGGAAAC | GAAAGCACGGTCTAAGGCAGTAG |
| Mouse | *Reg3g* | TTCCTGTCCTCCATGATCAAAA | CATCCACCTCTGTTGGGTTCA |
| Mouse | *Reg4* | AGAAGCAATTATGGCAGTGGACT | TGAGAGGGAAGTGGGAAGAGTTT |
| Mouse | *Zo-1* | TTCCAGAACCGAAACCTGTGTAT | CAGTTTCATGCTGGGCCTAAGTA |
| Human | *β-ACTIN* | CTCCATCCTGGCCTCGCTGT | GCTGTCACCTTCACCGTTCC |
| Human | *CLDN2* | TTCAGCAAGCCTTCCTTCTAGAG | CTCTTGCTCCTTGAACACCTCTA |
| Human | *GAPDH* | GGTGAAGGTTCGGTGAGTCAAC | CAAAGTTGTCATGGATGACACC |
| Human | *REG4* | CACGACCCACAGAAGAGGCAGC | GGCGCTTGTTGCATTCGTTGCT |
| Human | *PPARG* | AGATCATTTACACAATGCTGGC | TAAAGTCACCAAAAGGCTTTCG |
